# Domatinostat favors the immunotherapy response by modulating the tumor immune microenvironment (TIME)

**DOI:** 10.1186/s40425-019-0745-3

**Published:** 2019-11-08

**Authors:** Anne Catherine Bretz, Ulrike Parnitzke, Kerstin Kronthaler, Tobias Dreker, René Bartz, Frank Hermann, Astrid Ammendola, Tanja Wulff, Svetlana Hamm

**Affiliations:** 4SC AG, Fraunhoferstr. 22, 82152 Planegg-Martinsried, Germany

**Keywords:** Domatinostat, HDAC, Immunotherapy, Checkpoint inhibitor, Tumor immune microenvironment, IFN-γ signature, PD-1 blockade response signature

## Abstract

**Background:**

The efficacy of PD-(L)1 blockade depends on the composition of the tumor immune microenvironment (TIME) and is generally higher in tumors with pre-existing cytotoxic T cells (CTL) than in those with low CTL numbers. Nonetheless, a significant proportion of patients with pre-existing immunity fail to respond, indicating a therapeutic potential for combining PD-(L)1 blockade with additional immunomodulatory agents in both CTL-high and -low immune phenotypes. Here, we evaluated domatinostat (4SC-202), a class I-selective histone deacetylase (HDAC) inhibitor, for its effect on the TIME and its antitumoral efficacy using syngeneic mouse models with CTL-high or CTL-low tumors.

**Methods:**

Domatinostat was evaluated in PD-1 blockade-insensitive CTL-low (CT26) and CTL-high (C38) syngeneic models alone and in combination with different immune-inhibitory and -stimulatory approaches. Effects on the immunophenotype were assessed via flow cytometry and RNA-seq analyses. The changes in RNA-seq-based immune signatures determined in a murine setting were investigated in patient samples from the first-dose cohort of the SENSITIZE trial (NCT03278665) evaluating domatinostat combined with pembrolizumab in advanced-stage melanoma patients refractory/nonresponding to PD-1 blockade.

**Results:**

Domatinostat increased the expression of antigen-presenting machinery (APM) genes and MHC class I and II molecules, along with CTL infiltration, in tumors of both immune phenotypes. In combination with PD-(L)1 blockade, domatinostat augmented antitumor effects substantially above the effects of single-agent therapies, displaying greater benefit in tumors with pre-existing CTLs. In this setting, the combination of domatinostat with agonistic anti-4-1BB or both PD-1 and LAG3 blockade further increased the antitumor efficacy.

In CTL-low tumors, domatinostat enhanced the expression of genes known to reinforce immune responses against tumors. Specifically, domatinostat increased the expression of *Ifng* and genes associated with responses to pembrolizumab and nivolumab.

Clinically, these findings were confirmed in patients with advanced melanoma treated with domatinostat for 14 days, who demonstrated elevated expression of APM and MHC genes, the *IFNG* gene, and the IFN-γ and pembrolizumab response signatures in individual tumor samples.

**Conclusion:**

In summary, these data suggest a promising potential of domatinostat in combination with immunotherapy to improve the outcome of refractory cancer patients.

## Background

Immunotherapies targeting programmed cell death protein-1 (PD-1) and programmed cell death ligand-1 (PD-L1) checkpoints elicit durable antitumoral effects in multiple cancer indications. Objective response rates of 20–30% in urothelial cancer and head and neck squamous cell carcinoma and 50–60% in melanoma and Merkel cell carcinoma have been achieved in treatment-naïve advanced-disease patients but were generally lower in pretreated patients [1, 2]. To explain the wide variation in responses to checkpoint inhibition, pharmacodynamic data obtained in various checkpoint inhibitor trials were analyzed, and the concept of a tumor immunity continuum was developed, differentiating between inflamed and noninflamed tumors [3]. Inflamed tumors are characterized by the presence of tumor-infiltrating CD8^+^ T cells, increased IFN-γ signaling, expression of PD-L1, and high tumor mutational burden (TMB). Noninflamed tumors are immunologically ignorant, are poorly infiltrated by lymphocytes, and rarely express PD-L1. In-between, there are tumors that, although immunogenic, show increased influence of immunosuppressive stroma, myeloid-derived suppressor cells (MDSCs) or M2 macrophages, each of which suppresses T cell activation within the tumor immune microenvironment (TIME) or prevents infiltration of T cells into the tumor (reviewed in [3, 4]).

Clinical responses to PD-(L)1 blockade correlate with the presence of intratumoral T cells [5, 6]. Accordingly, tumors nonresponsive to anti-PD-(L)1 therapy are either deficient of T cells or, if T cell-inflamed, comprise mainly T cells of an exhausted phenotype, experience immunosuppression by myeloid cells, or evade cytotoxic T cell recognition by downregulating antigen presentation [7–9]. Administration of anti-PD-(L)1 monotherapy to these patients appears to be ineffective, indicating the need to combine PD-(L)1 blockade with additional immunomodulating drugs.

HDAC inhibitors (HDACis) are epigenetic modifiers known to have pleiotropic effects that increase immune responses by enhancing expression of cancer-germline antigens (CGA), MHC class I and II molecules (MHC-I and -II), components of the antigen-processing machinery (APM), and T cell-recruiting chemokines [10–15]. Inhibition of class I HDACs is associated with reduced number and immunosuppressive function of MDSCs and regulatory T cells (Tregs) [16, 17]. In murine models treated with combinations of HDACi with PD-(L)1 blockade, antitumor activity was superior to single-agent therapy [14, 18–20]; however, the mechanisms associated with these effects, particularly in tumors of different immunophenotypes, have not been fully elucidated. Although epigenetic drugs are being evaluated in combination with immunotherapy in several clinical trials, translational data on the immunomodulatory effects of class I-selective HDACis are scarce. In breast cancer patients, the number of peripheral MDSCs was significantly reduced upon treatment with the HDACi entinostat and the aromatase inhibitor exemestane [21].

Here, we studied the immunomodulatory effects of the class I-selective oral HDACi domatinostat (4SC-202). Domatinostat was previously tested in 24 patients with advanced hematological malignancies (phase I trial NCT01344707). Signs of anticancer activity were observed, including one patient with a complete response, one patient with a partial response and 18 patients with disease stabilization as the best overall response. Domatinostat was well tolerated, showing an acceptable safety profile [22].

To characterize the immune-related effects of domatinostat, two mouse syngeneic tumor models with low intrinsic response to checkpoint therapy and different levels of T cell infiltration were analyzed for immunologic changes in the TIME and antitumor activity. In both tumor models, domatinostat increased the number of intratumoral cytotoxic CD8^+^ T cells (cytotoxic T lymphocytes, CTLs), with the relative effect being more pronounced in tumors with low levels of pre-existing CTLs. In CTL-low tumors, domatinostat substantially induced the expression of *Ifng*, IFN-γ response genes and the PD-1 blockade response signature. In CTL-high tumors, domatinostat significantly increased CTLs expressing activation and proliferation markers, even within the PD-1/LAG3-double-positive CTL subpopulation.

In combination with PD-(L)1 blockade, domatinostat was able to significantly increase tumor response rates and survival of animals, particularly in tumors with high CTL levels. Likewise, double blockade of the inhibitory immune checkpoint receptors PD-1 and LAG3 or agonistic targeting of the costimulatory receptor 4-1BB augmented the antitumor effects of domatinostat in the CTL-high in vivo model.

Gene expression analysis of patient-derived melanoma biopsies after 14 days of domatinostat treatment (ongoing phase I/II trial NCT03278665) demonstrated increased expression of *IFNG*, the 10-gene IFN-γ signature, the pembrolizumab response signature, and APM/MHC genes and increased immune cytolytic activity scores in individual samples compared with baseline.

In summary, our data provide mechanistic insights into the immunomodulatory effects of domatinostat in cancer, supporting further clinical development of domatinostat in combination with immunotherapy.

## Methods

### In vivo mouse models

Animal housing and experimental procedures were performed in accordance with French and European Regulations and the NRC Guide for the Care and Use of Laboratory Animals. Female BALB/c mice (BALB/cByJ, Charles River) were subcutaneously (s.c.) injected into the right flank with 1 × 10^6^ CT-26 cells (ATCC) for tumor induction. For the immunocompromised model, BALB/c nude mice (CByJ. Cg-Foxn1nu/J, Charles River) were irradiated with a γ-source (whole-body irradiation, 2 Gy, ^60^Co) 24 h before CT26 cell engraftment. Colon C38 adenocarcinoma tumors were induced by grafting C38 tumor fragments (DCTD Tumor Repository, NCI) s.c. onto the right flank of female C57BL/6 J mice (Janvier). Treatment schedules were initiated when tumors reached a mean volume of 70–200 mm^3^. The length and width of tumors were measured twice a week with calipers, and tumor volumes were estimated by the formula: tumor volume = (width^2^ x length)/2. At necropsy, tumors were collected for further analyses as described below.

Domatinostat (CAS 1186222–89-8, provided by 4SC AG) was administered orally (p.o.) at 20 mg/kg twice daily (=40 mg/kg daily) or 60 mg/kg once daily for 12–14 days (CT26) or up to 24 days (C38). Antibodies (Bioxcell) were injected intraperitoneally (i.p.) at 10 mg/kg as scheduled: anti-PD-1 (RMP1–14, BE0146) twice weekly for two weeks, anti-PD-L1 (10F.9G2, BE0101) every three days for eight injections, and anti-LAG3 (C9B7W, BE0174) and anti-4-1BB (CD137, LOB12.3, BE0169) every three days for four injections.

### Immunohistochemistry (IHC)

Formalin-fixed, paraffin-embedded (FFPE) tissue sections were stained for CD3 and CD8 on a Bond RX Autostainer (Leica). Antigen retrieval was performed in EDTA (pH 9.0) at 100 °C for 20 min. CD3 and CD8 antibodies (Additional file 1) were incubated at RT for 60 min (1:100,1:400) and detected with ImmPACT Red Alkaline Phosphatase and DAB Peroxidase substrate kits (Vector), respectively.

### Flow cytometry analysis of tumor samples

Tumor samples were mechanically dissociated and dissolved in staining buffer (PBS, 0.2% BSA, 0.02% NaN_3_). For analysis of peripheral blood, red blood cells were lysed in lysing buffer (BD Biosciences). FcR blocking reagent (Miltenyi Biotec) was added, and each sample was incubated with antibodies in staining buffer rinsing solution plus 0.5% BSA (Miltenyi Biotec) according to the supplier’s instructions (Additional file 1). For intracellular labeling, a staining buffer set (Miltenyi Biotec) was used. After washing, cells were resuspended in PKH26 reference microbead solution (Sigma-Aldrich) and analyzed using multicolor flow cytometry (CyFlow space, Sysmex; LSR II or Fortessa X20, both BD Biosciences). Quantitative expression data of selected markers are presented as geometric mean fluorescence intensity (gMFI), cell type frequencies as the percentage of viable singlet cells of a defined population.

### Gene expression analysis

#### RNA isolation

Total RNA from cell culture (A375, CT26) or fresh-frozen tumor samples (CT26) was isolated with the RNeasy Mini Kit (Qiagen). RNA from FFPE-tissue sections (patient tumor samples) was isolated with the AllPrep DNA/RNA FFPE Kit (Qiagen). A DNase digestion step was included. RNA concentration and integrity were assessed with the Experion RNA StdSens kit (Bio-Rad) or the Bioanalyzer RNA 6000 Nano chips (Agilent Technologies).

#### RNA sequencing (RNA-seq)

Libraries were prepared using Illumina TruSeq Stranded mRNA (A375, CT26) or TruSeq RNA Exome technology (FFPE tissue) and were quality-controlled with DNA 1000 chips (Agilent Technologies). Multiplexed samples were pooled and quantified using the Qubit dsDNA HS Assay (Invitrogen). RNA sequencing was performed on the Illumina NextSeq500 next-generation sequencing system with 1 × 75 bp single-end or, for FFPE samples, 2 × 75 bp paired-end high-output runs.

Primary image processing, data analysis and demultiplexing were carried out with Real-Time Analysis software and bcl2fastq. Technical quality parameters were evaluated with the Illumina Sequence Analysis Viewer. High-quality sequenced reads were imported into the CLC Genomics Workbench (Qiagen) and aligned to the mouse (GRCm38.p3 C57BL/6, NCBI) or human reference genome (GRCh38.p7, NCBI). Absolute gene expression was quantified by the number of reads (counts) per gene and was transformed to normalized transcripts per million (TPM) values.

Gene expression for selected gene sets was visualized with heatmaps using morpheus.R (https://software.broadinstitute.org/morpheus) and log2-transformed TPM + 0.001 values. Signature scores were calculated for each sample from the mean log2(TPM + 0.001) values of the corresponding genes.

Differential gene expression (DGE) was evaluated by DESeq2 [23] using unique gene reads (counts) per gene, comparing the expression between domatinostat and control groups with default parameters (parametric dispersion). DGE is displayed as log2-fold change (FC) with adjusted *P-*values for multiple testing (Benjamini-Hochberg correction). Gene set enrichment analysis (GSEA) was performed with normalized expression data and default parameters [24].

#### SENSITIZE clinical trial (NCT03278665)

SENSITIZE is a phase Ib/II open-label, multicenter trial evaluating the safety and preliminary efficacy of domatinostat combined with pembrolizumab in patients with advanced (unresectable or metastatic) cutaneous melanoma that is primary refractory or nonresponding to anti-PD-1 therapy. The trial received independent ethics committee approval and is conducted in accordance with the Declaration of Helsinki, the International Conference on Harmonization, Guidance on Good Clinical Practice, and all regulatory requirements regarding conduct of human clinical trials. Written informed consent was obtained from each patient prior to any trial procedure. For our analyses, biopsies of different cutaneous, subcutaneous or visceral metastases were obtained from patients of the first-dose cohort before (screen) and after the first priming cycle (14 days, C01D14) of domatinostat monotherapy (100 mg once daily).

#### Statistics

Quantitative data are presented as mean ± standard deviation or as a box plot (whiskers: min to max) with individual data points. Statistics were performed with GraphPad Prism. For two-group comparisons, significance (two-tailed *P*-value) was determined by the nonparametric Mann-Whitney test. For more than two groups, the nonparametric Kruskal-Wallis test with Dunn’s multiple comparison test was applied. Time to event (tumor volume of 1500 mm^3^) was calculated by using a linear interpolation between the closest values (log scale). Time to event-free survival was analyzed by Kaplan-Meier plots and statistically evaluated by a pairwise log-rank (Mantel-Cox) test of treatment versus vehicle. Animals taken out for FACS analysis before the event were censored. Correlation analysis was performed by the Pearson method. *P*-values were categorized as follows and are listed in each figure: ns (not significant): *P* > 0.05; *: *P* < 0.05; **: *P* < 0.01; ***: *P* < 0.001; ****: *P* < 0.0001.

## Results

### Domatinostat increases tumor control and inflammation in CTL-low CT26 tumors resistant to PD-(L)1 blockade

Domatinostat is a class I-selective HDACi (Additional file 2: Figure S1a). During in vitro analysis using human melanoma and murine CT26 cells, domatinostat increased the expression of CGAs and MHC molecules known to enhance the immunogenicity and recognizability of tumor cells (Additional file 2: Figure S1b-f). Therefore, domatinostat was tested in vivo using the mouse syngeneic CT26 model. CT26 tumors are known to have high TMB [25], but they harbor only low numbers of CTLs (~ 0.1%; Additional file 2: Figure S2a), thus representing noninflamed, CTL-low tumors.

**Fig. 1:**
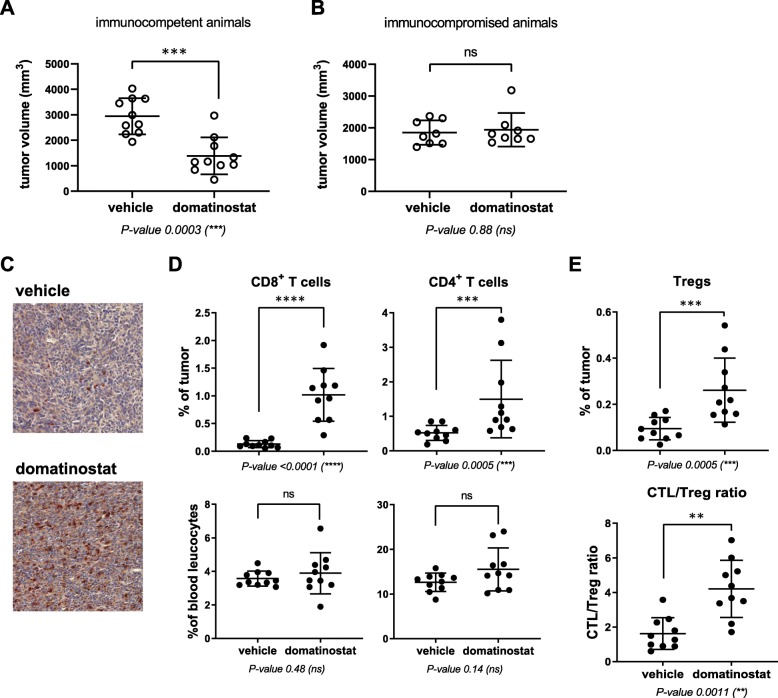
Domatinostat decreases tumor volume and induces strong CTL infiltration in the CTL-low CT26 tumor model. CT26 cells (1 × 10^6^) were inoculated s.c. into immunocompetent (**a**, **c**-**e**; *n* = 10 per group) and immunocompromised (b; *n* = 8 per group) BALB/c mice; when tumor volumes reached 150 mm^3^, animals were treated with domatinostat (20 mg/kg twice daily) or vehicle; after the end of treatment, tumors were harvested for flow cytometry and IHC. **a**, **b**, Tumor volumes in immunocompetent (**a**) and immunocompromised animals (**b**). **c**, CD3-AP (red) and CD8-DAB (brown) double IHC staining of the tumor core. **d**, Proportion of CD8^+^ and CD4^+^ T cells in tumors (upper panel) and blood (lower panel). **e**, Proportion of Tregs and CTL/Treg ratio in tumors. ***a****,* ***b****,* ***d****,* ***e***, Mean ± SD showing all data points; P-values: Mann-Whitney test, two-tailed, **, P < 0.01; ***, P < 0.001; ****, P < 0.0001; ns, not significant

Treatment with domatinostat decreased tumor volume by 53% in immunocompetent but not in immunocompromised mice (Fig. 1a,b), suggesting an immune-dependent antitumoral mechanism of action for domatinostat. Immunocompetent mice on domatinostat treatment showed an increased number of CTLs within the tumor core (Fig. 1c). Detailed analysis of tumor cell populations revealed an ~ 8-fold increase in CTLs and an ~ 3-fold increase in CD4^+^ T cells following domatinostat treatment. These increases were restricted to the TIME; cell populations in peripheral blood remained unchanged (Fig. 1d). Despite elevations in Tregs, the CTL/Treg ratio was significantly increased on domatinostat versus control (Fig. 1e), favoring antitumor immune responses [26].

**Fig. 2:**
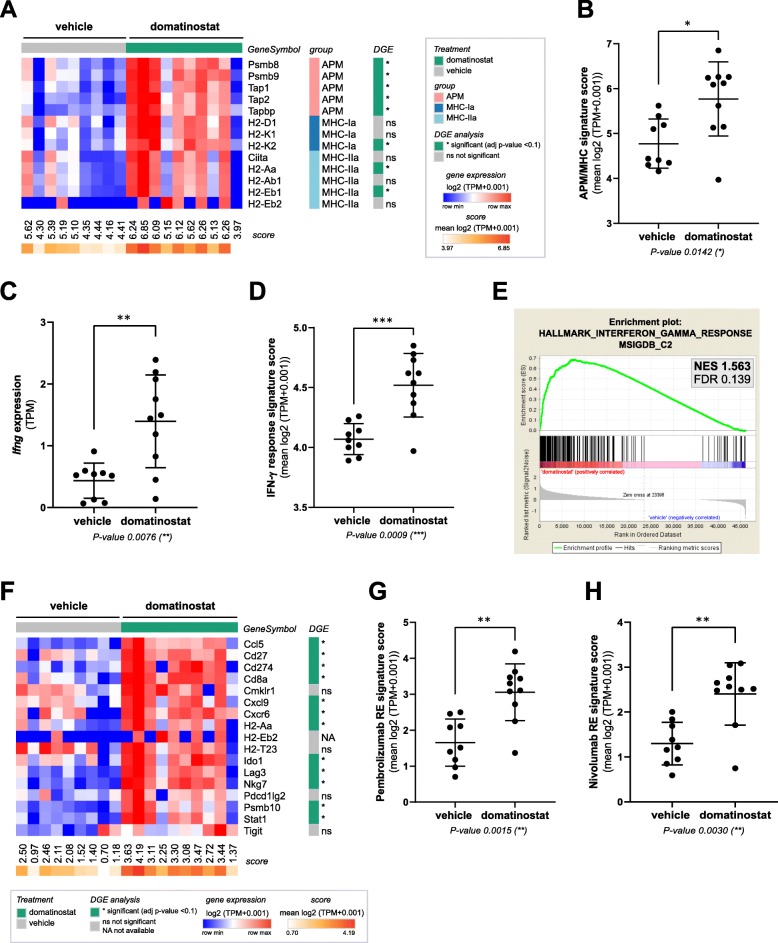
Domatinostat increases gene expression signatures correlated with the clinical benefit of PD-1 blockade. CT26 tumor model (n = 10 per group) as in Fig. 1; end-of-treatment tumors were analyzed for gene expression by RNA-seq. **a**, Heatmap of antigen-processing machinery (APM) and major histocompatibility complex (MHC) class I and II gene expression with scores per sample. **b**, APM/MHC signature score based on (**a**). **c**, Ifng gene expression. **d**, IFN-γ response signature score (MSigDB hallmark gene set). **e**, Gene set enrichment analysis (GSEA) plot for the correlation of domatinostat-regulated gene expression with the IFN-γ response signature (MSigDB). NES: normalized enrichment score; FDR: false discovery rate. **f**, Heatmap of pembrolizumab response signature gene expression (adapted from Ayer’s T cell inflamed signature) [5]. **g**, Pembrolizumab response (RE) signature score based on (**f**). **h**, Nivolumab response (RE) signature score [27]. ***b****,* ***c****,* ***d****,* ***g****,* ***h***, Mean ± SD showing all data points; signature scores were calculated by mean log2(TPM + 0.001) of their respective member genes; P-values: Mann-Whitney test, two-tailed. *, P < 0.05; **, P < 0.01; ***, P < 0.001; ns, not significant. TPM, transcripts per million; DGE, differential gene expression

Gene expression analysis of CT26 tumors revealed the induction of a plethora of immune-related pathways by domatinostat (Additional file 2: Figure S3a). In particular, treatment with domatinostat resulted in upregulation of APM and MHC-I and -II genes as well as proinflammatory *Ifng* and IFN-γ response genes (Fig. 2a-e; Additional file 2: Figure S3b). Furthermore, domatinostat increased the expression of genes positively associated with responses to the PD-1 antibodies pembrolizumab (adapted from [5]; Fig. 2f,g) and nivolumab (adapted from [27]; Fig. 2h). All gene expression scores showed a highly significant positive correlation. In addition, the decrease in tumor volumes upon treatment with domatinostat significantly correlated with increases in intratumoral CTLs, *Ifng* expression and all tested scores (Additional file 2: Figure S3c).

**Fig. 3:**
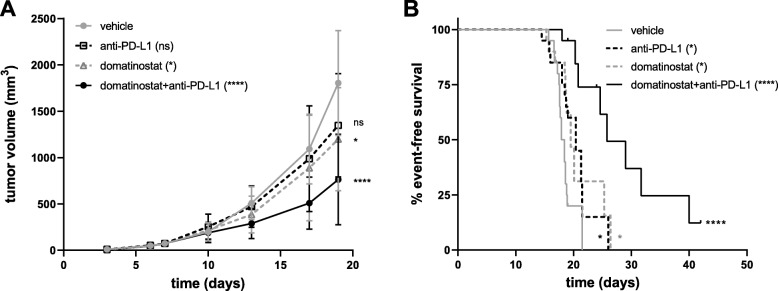
Domatinostat synergizes with PD-L1 blockade to prolong survival. CT26 tumor model as in Fig. 1; animals were treated with 20 mg/kg domatinostat twice daily, 10 mg/kg anti-PD-L1 antibody twice a week or the combination of both and were compared with vehicle-treated animals (*n* = 20 per group). **a**, Tumor volumes (mean ± SD) over time. **b**, Kaplan-Meier event-free survival plots. An event was defined as a tumor volume of 1500 mm^3^. P-values: a, Kruskal-Wallis test (d19); Dunn’s multiple comparison to vehicle. b, Log-rank (Mantel-Cox) test, comparison to vehicle. *, P < 0.05; ****, P < 0.0001; ns, not significant

The obtained in vivo data of domatinostat encouraged combination therapy with PD-(L)1 blockade. In the CT26 tumor model, PD-L1 or PD-1 antibodies alone hardly affected tumor growth (Fig. 3a and Additional file 2: Figure S2b, respectively), whereas domatinostat significantly reduced tumor volume by 34% compared with the control (Fig. 3a). In combination with anti-PD-L1, domatinostat further reduced tumor volumes, resulting in prolonged event-free survival of the animals (event defined as tumor volume of 1500 mm^3^; Fig. 3b). Furthermore, all event-free animals in the combination group (10%) were completely tumor-free at the end of the study.

### Domatinostat increases both the number and effector function of T cells and enhances the anticancer effects of PD-1 blockade in CTL-high C38 tumors

**Fig. 4:**
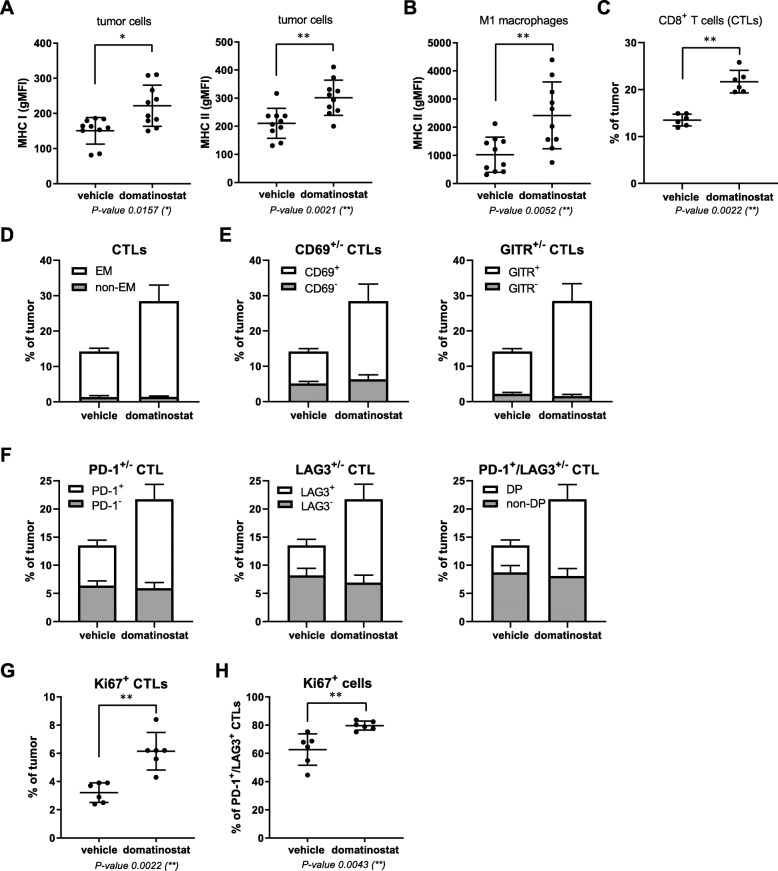
In CTL-high C38 tumors, domatinostat treatment results in activated effector CTL populations expressing PD-1/LAG3. C38 tumor fragments were inoculated s.c. into C57BL/6 J mice; when tumor volumes reached 150 mm^3^, animals were treated with 20 mg/kg domatinostat or vehicle twice daily; tumors were harvested for analysis of cell populations by flow cytometry after 9 (**c**-**h**, *n* = 6) or 18 treatment days (**a**, **b**, n = 10). **a**, MHC class I and II expression on tumor cells (CD45^−^). **b**, MHC class II expression on M1 macrophages (CD45^+^CD3^−^CD11b^+^CD38^+^). **c**, Proportion of CTLs (CD3^+^CD8^+^) within tumors. **d**-**h**, Characterization of intratumoral CTLs: proportions of the effector memory (EM, CD44^+^CD62L^−^) (**d**), CD69^+^ and GITR^+^ (**e**), PD-1^+^, LAG3^+^ and PD-1^+^/LAG3^+^ double-positive (DP) (**f**) and Ki67^+^ CTLs (**g**) and of Ki67^+^ cells within the PD-1^+^/LAG3^+^ CTL population (**h**). ***a****-****c****,* ***g****,* ***h****, Mean ± SD showing all data points; gMFI, geometric mean fluorescence intensity.* ***d****-****f***, Mean + SD shown in stacked bars. P-value: Mann-Whitney test, two-tailed. *, P < 0.05; **, P < 0.01

**Fig. 5:**
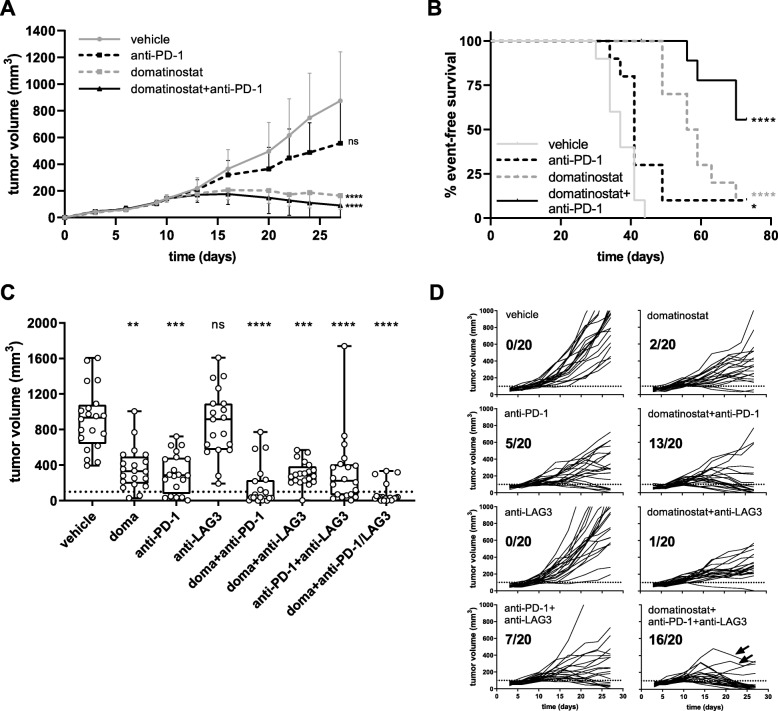
Combination therapy of domatinostat with PD-1 and LAG3 blockade significantly increases antitumoral responses. C38 tumor model as in Fig. 4; animals were treated with 60 mg/kg domatinostat once daily (**a**, **b**) or 20 mg/kg domatinostat twice daily (**c**, **d**); anti-PD-1 and anti-LAG3 antibodies were administered at 10 mg/kg as detailed in the Methods (n = 20 per group). **a**, Tumor volumes (mean + SD) over time. **b**, Kaplan-Meier event-free survival plots. An event was defined as a tumor volume of 1500 mm^3^. End of study was day 70. **c**, Tumor volumes at day 27; response was defined as tumor regression below a volume of 100 mm^3^ (dotted line). **d**, Changes in tumor volumes over time for each individual animal and the number of responding animals out of the total for treatment regimens corresponding to (**c**); arrows indicate two animals with an incipient tumor regression after initial progress. ***c****, Box and whiskers (min, max) showing all points.* ***a****,* ***c****, P-value: Kruskal-Wallis test; Dunn’s multiple comparison (d27) to vehicle.* ***b***, P-value: log-rank (Mantel-Cox) test, comparison to vehicle. *, P < 0.05; **, P < 0.01; ***, P < 0.001; ****, P < 0.0001; ns, not significant

Melanoma often presents as inflamed tumors with high numbers of CTLs [6, 28]. Despite a high T cell-inflamed gene expression profile, the percentage of nonresponders to PD-1 blockade is still > 40% [6]. Likewise, the mouse syngeneic C38 tumor model comprises high numbers of CTLs (~ 14%; Fig. 4c: vehicle) but a response rate to PD-1 blockade of only 10–25% (Fig. 5b,d: anti-PD-1). We thus used C38 cells to evaluate the effects of domatinostat on inflamed tumors.

Similar to CT26, in vivo treatment with domatinostat increased the expression of MHC-I and -II molecules on C38 tumor cells and of MHC-II molecules on M1 macrophages (Fig. 4a,b). Furthermore, the number of CTLs within the tumors rose to 22% upon domatinostat treatment (Fig. 4c). Most of these CTLs were of the effector memory (EM) phenotype (CD44^+^CD62L^−^) and expressed the activation markers CD69 and GITR as well as the inhibitory marker PD-1, LAG3 or both (Fig. 4d-f). These inhibitory receptors are upregulated as a negative feedback mechanism limiting T cell effector function following an antigen-specific stimulation of T cells [29]. Hence, our findings suggest a role for domatinostat in the antigen-specific activation of CTLs.

Domatinostat almost doubled the number of CTLs expressing the proliferation marker Ki67 to approximately 6% (Fig. 4g). Interestingly, within the subpopulation of PD-1/LAG3-double-positive CTLs, domatinostat significantly increased Ki67-positive cells from 60 to 80% (Fig. 4h).

In the C38 tumor model, high antitumor activity was observed for domatinostat and domatinostat+anti-PD-1 combination therapy, whereas anti-PD-1 alone did not significantly reduce tumor volumes (Fig. 5a). Treatment with domatinostat alone prolonged the median event-free survival from 37 days (vehicle) to 57.5 days. The rate of event-free survival was 10% for domatinostat and anti-PD-1 monotherapies. Domatinostat in combination with anti-PD-1 substantially increased the event-free survival, not reaching the median at the end of the study, and resulted in a significantly better tumor control, with 56% of event-free animals (Fig. 5b). Moreover, these animals were completely tumor-free at the end of the study.

### Combining domatinostat with PD-1 and LAG3 antibodies achieves superior antitumor responses in CTL-high tumors

In the CTL-high C38 tumor model, domatinostat increased the expression of MHC-II molecules not only on tumor cells and M1 macrophages (Fig. 4a,b; as outlined above) but also on Ly6C^+^ or Ly6G^+^ myeloid cells (Additional file 2: Figure S4). Upregulation of MHC-II on macrophages may promote CD4^+^ T cell priming and reduce the immunosuppressive activity of Ly6C^+^ or Ly6G^+^ myeloid cells [30]. However, MHC-II molecules are ligands of LAG3, whose engagement on T cells is known to limit the T cell attack on tumor cells [29]. We therefore hypothesized that the addition of LAG3 blockade to the combination of domatinostat and anti-PD-1 would further increase the antitumor effects.

Indeed, triple combination therapy with domatinostat, anti-PD-1 and anti-LAG3 showed the highest antitumor activity of the treatment regimens tested (Fig. 5c,d). Previous findings were confirmed, with response rates (defined as tumor regression below a volume of 100 mm^3^) of 10, 25 and 65% for domatinostat, anti-PD-1 and their combination, respectively. Treatment with anti-LAG3 alone was inefficacious and only slightly increased the antitumoral efficacy in combination with anti-PD-1 (response rate: 35%, Fig. 5d). However, in triple combination therapy, responses were observed in 16/20 animals (80%), and the tumors of two additional animals started to regress after initial progression (Fig. 5d: arrows).

### Synergy of domatinostat and the agonistic 4-1BB antibody in CTL-high tumors

Since T cell activity can be modulated by inhibitory and costimulatory signals, agonizing costimulatory receptors is another approach to boost T cell responses (reviewed in [31]). 4-1BB (CD137) is a costimulatory receptor expressed on activated T cells, triggering enhanced effector functions. In exhausted CTLs, 4-1BB signaling is able to restore cytotoxic capacities [32].

**Fig. 6:**
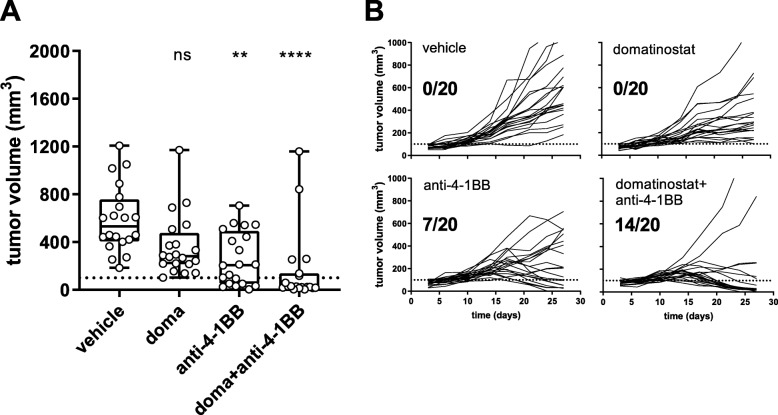
Combination therapy of domatinostat with the agonistic anti-4-1BB antibody significantly increases antitumoral responses. C38 tumor model as in Figs. 4 and 5; animals were treated with 20 mg/kg domatinostat twice daily; agonistic anti-4-1BB antibody was administered at 10 mg/kg as detailed in the Methods (n = 20 per group). **a**, Tumor volumes at day 27; response was defined as tumor regression below a volume of 100 mm^3^ (dotted line). **b**, Changes in tumor volume over time for each individual animal and for responding animals out of the total for each treatment regimen listed in (**a**). ***a***, Box and whiskers (min, max) showing all points, P-value: Kruskal-Wallis test; Dunn’s multiple comparison (d27) to vehicle. *, P < 0.05; **, P < 0.01; ***, P < 0.001; ****, P < 0.0001; ns, not significant

In the CTL-high C38 model, the combination of domatinostat and the agonistic 4-1BB antibody significantly decreased tumor volumes and led to tumor responses in 14/20 animals (70%). Of note, monotherapy with anti-4-1BB achieved responses in 7/20 animals (35%), whereas domatinostat alone did not significantly reduce tumor volumes in this experiment (Fig. 6).

### Domatinostat upregulates genes associated with the response to immunotherapy in biopsies of domatinostat-treated melanoma patients

Domatinostat in combination with pembrolizumab is currently being evaluated in patients with advanced cutaneous melanoma primary refractory or nonresponding to anti-PD-1 therapy (SENSITIZE trial: NCT03278665; completion expected Dec 2020). Different doses and regimens of domatinostat are applied. In all patients, treatment starts with domatinostat for 14 days prior to combination therapy. Biopsies were collected at screening (pretreatment/baseline) and day 14 to analyze the effects of domatinostat on the TIME. Here, the expression of immune-related gene sets was analyzed in biopsies from 6/10 patients of the first-dose cohort receiving 100 mg domatinostat once daily (patient characteristics: Additional file 2: Table S1). Samples included tissues from different cutaneous, subcutaneous or visceral metastases. Due to the low sample number, heterogeneity of tumor lesions and presumed suboptimal dosing of domatinostat, the data are considered exploratory.

**Fig. 7:**
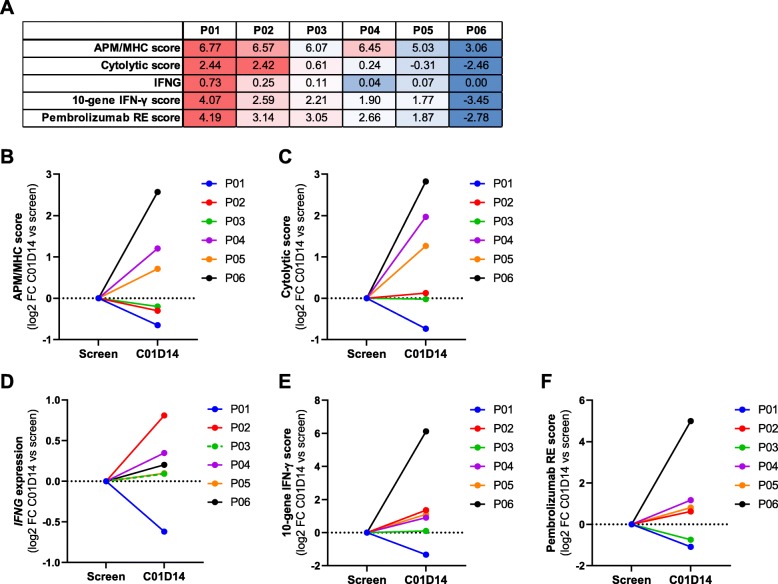
Domatinostat increases IFN-γ, APM/MHC and PD-1 therapy response scores in biopsies of domatinostat-treated melanoma patients. Six patients (P01-P06) with advanced cutaneous melanoma treated with 100 mg domatinostat once daily in a phase I/II clinical trial (SENSITIZE trial: NCT03278665) were subjected to biopsy of tumor lesions before (screen, baseline) and after 14 days of treatment (C01D14) for analysis of gene expression (RNA-seq). **a**, Baseline expression scores (mean log2(TPM + 0.001)) for selected gene sets per patient. **b**-**f**, Expression changes after 14 days of domatinostat monotherapy (C01D14) shown as log2 fold change (FC) from the baseline score for APM/MHC genes (**b**), cytolytic activity genes (**c**), IFNG (**d**), the 10-gene IFN-γ-related signature [5] (**e**) and the pembrolizumab response (RE) signature [5] (**f**)

Gene expression analysis of pretreatment samples revealed a considerable difference in the overall number and composition of immune cells. In the tumors, CTLs were present in distinct (patients P01, P02, P03) or low proportions (P04) or were completely absent (P05, P06). In the biopsy of patient P06, the overall number of immune cells was very low (Additional file 2: Figure S6a). Analysis of immune-related gene sets in pretreatment samples confirmed the CTL-based ranking, with P01 demonstrating the highest and P06 the lowest expression level for all scores (Fig. 7a).

In line with our murine in vivo findings, the APM/MHC expression score increased upon domatinostat treatment in 3/6 patients (P04, P05, P06). This score was barely changed from baseline in 2/6 patients (P02, P03) and was slightly reduced in patient P01 (Fig. 7b, heatmap: Additional file 2: Figure S6b). Similar results were obtained for the immune cytolytic activity score [33], reflecting the functional efficacy of cytotoxic T cells based on the expression of CD8A, CD8B, granzymes and perforin genes (Fig. 7c). These genes were shown to be upregulated upon CD8^+^ T cell activation and correlated with clinical responses to CTLA-4 and PD-L1 [33, 34]. Furthermore, treatment with domatinostat slightly increased *IFNG* gene expression in 5/6 patients (Fig. 7d). The scores of the 10-gene IFN-γ-related signature and the pembrolizumab response signature [5] were enhanced in 4/6 patients each (patients P02, P04, P05, P06; Fig. 7e,f; corresponding heatmaps: Additional file 2: Figure S6c,d). Despite a slight increase in *IFNG* gene expression in patient P03, the IFN-γ-related signature remained unchanged, and the pembrolizumab response signature decreased. Patient P01 exhibited reductions in *IFNG* and all expression scores after 14 days of domatinostat therapy. Of note, this patient already had the highest baseline immune scores of all patients. Conversely, patient P06, with the lowest baseline expression, showed the highest upregulation of gene expression in all scores tested.

In summary, gene expression analysis of tumor biopsies from patients treated with domatinostat for 14 days revealed changes in the TIME known to support responses to immune checkpoint blockade in melanoma patients.

## Discussion

HDACis are known to upregulate the expression of CGA, MHC-I and -II, APM and chemokine genes, which are associated with enhanced immunogenicity and improved recognition of tumor cells by T cells [10–15]. In addition, some HDACis were shown to reduce the number and function of immunosuppressive cells [16, 17]. Overall, HDACis induce changes in the TIME that support antitumoral immune responses [18–20] and may thus be ideal candidates for combination with cancer immunotherapies.

Domatinostat is a class I-selective HDACi currently in clinical development for the treatment of advanced cutaneous melanoma (NCT03278665) and gastrointestinal cancer (NCT03812796). In these trials, domatinostat is administered in combination with PD-1 and PD-L1 blockade, respectively. To characterize potential antitumor and immunologic effects, we performed a series of in vivo experiments with domatinostat alone and in combination with immunotherapies using mouse syngeneic tumor models of low intrinsic response to PD-(L)1 blockade and different levels of T cell infiltration.

T cell immunity requires the recognition of antigens. Tumor escape mechanisms thus comprise impaired tumor antigen expression, malfunctioning of the APM and/or surface presentation of peptides by MHC-I molecules, preventing recognition and binding of CTLs and subsequent destruction of tumor cells [35]. Domatinostat increased the expression of CGA, APM and MHC-I genes both in vitro and in vivo, addressing these particular resistance mechanisms. Interestingly, domatinostat also increased MHC-II molecules on tumor and immune cells in vivo. The expression of MHC-II and costimulatory molecules on tumor cells, particularly within a proinflammatory TIME, was shown to augment tumor-specific CTL and T-helper responses, leading to tumor rejection and protective long-term and memory immune responses [36]. In triple-negative breast cancer and colorectal carcinoma, MHC-II expression has correlated with a favorable prognosis of patients [37, 38]. In anti-PD-1-treated melanoma patients, MHC-II positivity in addition to ubiquitous MHC-I expression has been associated with CD4^+^ and CD8^+^ T cell infiltrates and has been predictive for the response to PD-1 blockade and overall survival [39]. Upregulation of MHC-II molecules by domatinostat may thus enhance antitumoral immune responses in vivo.

IFN-γ signaling supports antitumor immune responses in several ways. It can upregulate the expression of MHC molecules and APM in both tumor and immune cells [40], promote tumoricidal activity of macrophages [41], and be crucial for T and NK cell trafficking into tumors through induction of the chemokines CXCL10 and − 11 [42]. In melanoma patients, a 10-gene IFN-γ-related gene signature has been associated with response to PD-1 blockade and clinical benefit [5]. In CT26 tumors, treatment with domatinostat increased the expression of *Ifng* and IFN-γ response genes, which are known to enhance inflammation and support immune responses against tumor cells. While domatinostat directly affected APM/MHC genes, the upregulation of IFN-γ did not seem to be an immediate effect of domatinostat on *Ifng* gene expression. Although domatinostat increased intratumoral expression of *Ifng* and IFN-γ target genes in vivo, it neither upregulated their expression in the CT26 cell line nor induced IFN-γ in isolated peripheral blood mononuclear cells (PBMCs) in vitro (Additional file 2: Figure S7), suggesting an indirect effect. In CT26 tumors, elevated *Ifng* gene expression was associated with increased intratumoral CTLs, which may be the source of IFN-γ in vivo. The combination of domatinostat with IFN-γ in vitro resulted in a stronger upregulation of MHC-I than either agent alone (Additional file 2: Figure S1c,d), indicating a possible synergistic effect of domatinostat and IFN-γ on MHC expression in the TIME.

In vivo, domatinostat induced 8- and 1.6-fold increases in cytotoxic T cells, resulting in CTL levels of ~ 1 and 22% of the total tumor cells, in CTL-low CT26 and CTL-high C38 tumors, respectively. Despite high baseline CTL levels, C38 tumors have shown limited responses to PD-(L)1 blockade, suggesting impaired functionality of CTLs. T cells persistently exposed to inflammatory signals or antigenic stimulation are known to become exhausted over time [43]. T cell exhaustion is characterized by the coexpression of several inhibitory checkpoints, including PD-1 and LAG3. Indeed, a proportion of CTLs expressed PD-1, LAG3 or both, indicating previous antigen-specific activation and emerging exhaustion of T cells in our study. Since C38 fragments are used for passaging the tumor between animals, the cells might have reached a “mature” immune phenotype promoting T cell exhaustion in this model.

Although ~ 20% of CTLs were proliferating (3.2% Ki67^+^ within the 13.5% of total cells that were CTLs) in vehicle-treated C38 tumors, no tumor control was achieved. Domatinostat strongly increased the percentage of activated and proliferating CTLs without affecting the absolute number of nonactivated CTLs (non-EM, CD69^−^, GITR^−^, PD-1^−^, LAG3^−^) or the expression levels of the inhibitory receptors PD-1 and LAG3 on CTLs positive for these markers (Additional file 2: Figure S5a,b). While PD-1 blockade had no effects (Additional file 2: Figure S5c), domatinostat significantly increased the number of proliferating CTLs within the PD-1^+^/LAG3^+^ subpopulation, indicating a beneficial effect of domatinostat on the functionality of CTLs coexpressing these exhaustion markers.

Overall, domatinostat increased not only the overall number of CTLs but also the number of activated and proliferating CTLs of the EM phenotype. Domatinostat thus induced the generation of functional, tumor-specific T cells necessary for efficacious antitumor immune responses. Indeed, mean tumor volumes decreased significantly on domatinostat monotherapy compared with vehicle in both syngeneic mouse models.

The observed actions of domatinostat on the TIME together with the known mechanisms of resistance to immunotherapy indicate a high potential value of its combination with immune checkpoint blockade. This was further supported by the intratumoral upregulation of genes associated with responses to pembrolizumab [5] and nivolumab [27] in CT26 tumors treated with domatinostat. Therefore, different regimens were tested in vivo for their antitumor effects. The combination of domatinostat and PD-(L)1 blockade significantly prolonged survival in animals with CT26 and C38 tumors, with 10 and 56% of the animals being completely tumor-free at the end of the study, respectively. Combination therapy was thus efficacious in both tumor immunophenotypes, with a higher benefit in tumors with pre-existing CTLs.

In contrast to other cancer indications, the expression of MHC-II is correlated with poor prognosis in melanoma [44]. MHC-II is a ligand of the inhibitory checkpoint receptor LAG3, which is substantially expressed on melanoma-infiltrating T cells [45]. Domatinostat increased the expression of MHC-II on both tumor and immune cells, which is beneficial for CD4^+^ T cell priming in principle; however, interactions with the LAG3 receptor could subvert the CD4^+^ T cell response against the tumor [46]. Hence, we hypothesized that blocking LAG3 in addition to PD-1 blockade may increase tumor-specific T cell responses promoted by domatinostat. Indeed, the triple combination of domatinostat, anti-PD-1 and anti-LAG3 resulted in an increased response rate compared with the corresponding mono- or double therapies in the C38 tumor model.

In addition to the inhibitory receptors PD-1 and LAG3, the costimulatory receptor 4-1BB (CD137) is also highly expressed on exhausted T cells [32]. Stimulation of 4-1BB was shown to increase T cell responses and improve the antitumor effects of PD-1 blockade in vivo by improving T cell metabolic and respiratory capacities [47, 48]. In the C38 tumor model, the combination of domatinostat with an agonistic 4-1BB antibody doubled the tumor response rate over anti-4-1BB alone, further emphasizing the ability of domatinostat to increase the functional activity of CTLs.

Translational data on the immunologic effects of HDACis are scarce so far. Entinostat, like domatinostat a class I-selective HDACi, was shown to reduce the number and function of MDSCs in murine models in which combination therapies with checkpoint inhibitors were evaluated [48]. Based on this mode of action, peripheral MDSCs were evaluated in advanced breast cancer patients treated with a combination of entinostat and exemestane [21]. Blood samples collected after two weeks of therapy revealed significantly decreased MDSCs. These findings were confirmed in an ongoing trial administering entinostat to patients with advanced solid tumors [49]. Of note, following domatinostat treatment, we observed a slight reduction in MDSCs in CT26 tumors, but not in blood (Additional file 2: Figure S2c,d). To the best of our knowledge, there are no published data describing immunological changes in tumors of patients treated with class I-selective HDACis as monotherapy.

To characterize the effects of domatinostat in humans, biopsies from 6 patients with advanced melanoma (ongoing phase I/II trial SENSITIZE) were analyzed before and after 14 days of domatinostat therapy. Immune cell composition at baseline illustrated considerable heterogeneity between the patients. Nevertheless, clinical gene expression data of baseline versus treated tumor lesions confirmed our previous murine in vivo findings. Domatinostat increased *IFNG* expression in 5/6 patients, the scores for the 10-gene IFN-γ-related and pembrolizumab response signatures in 4/6 patients, and APM/MHC and cytolytic activity expression in 3/6 patients each. Of note, the patient with the lowest presence of immune cells in the pretreatment biopsy showed the highest upregulation of gene expression in all scores applied. The low number of samples does not allow statistically reliable interpretations. Nonetheless, although preliminary, the data obtained from patients treated with 100 mg domatinostat once daily in the first-dose cohort of the trial further support the development of domatinostat in combination with cancer immunotherapy.

## Conclusion

In summary, we have demonstrated that domatinostat increased both the overall number of CTLs and the number of functional cytotoxic T cells, which may effectively target tumor cells and ensure the induction of antitumor immune responses. In addition, domatinostat enhanced the expression of CGA, APM and MHC-I and -II genes both in vitro and in vivo. These effects may increase the immunogenicity of tumor cells and support the recognition of tumor cells by CD4^+^ and CD8^+^ T cells. By inducing *Ifng* and IFN-γ-related genes, domatinostat established a proinflammatory TIME, which is known to reinforce immune responses against tumor cells. Specifically, domatinostat induced the expression of genes associated with responses to pembrolizumab and nivolumab, further supporting the suitability of domatinostat for combination therapy with PD-1 blockade.

Domatinostat monotherapy exhibited antitumor activity in all in vivo systems analyzed. In combination with PD-(L)1 blockade, domatinostat augmented the antitumor effects substantially above the effects observed for single-agent therapies, with a greater benefit in tumors with pre-existing CTLs. In this setting, combination of domatinostat with the agonistic 4-1BB antibody or with both PD-1 and LAG3 blockade further increased the antitumor efficacy.

Based on the observed preliminary translational immunomodulatory effects of domatinostat, synergy with immune checkpoint inhibition may also be expected in patients. Since there is an urgent need to increase response rates and improve survival in patients on immunotherapy, combination with domatinostat is a potential treatment option for cancer patients.

## Supplementary information

**Additional file 1:** Supplementary methods. (DOCX 56 kb)

**Additional file 2: Figure S1.** In vitro effects of domatinostat on antigen presentation by human melanoma and murine CT26 cells. **Figure S2.** Immune cell profiling of mouse syngeneic CT26 tumors. **Figure S3.** In vivo effects of domatinostat on gene expression in murine syngeneic CT26 tumors. **Figure S4.** Domatinostat increases MHC class II expression in MDSCs in the syngeneic C38 tumor model. **Figure S5.** Phenotype of immune checkpoint-positive cells after domatinostat or anti-PD-1 treatment in the syngeneic C38 tumor model. **Figure S6.** Gene expression analysis of patient-derived domatinostat-treated melanoma biopsies. **Figure S7.** Domatinostat has no direct effect on IFN-γ expression. **Table S1.** Patient characteristics (SENSITIZE, cohort 1). (DOCX 2645 kb)

## Data Availability

The datasets used and/or analyzed during the current study are available from the corresponding author on reasonable request.

## Abbreviations

APM: Antigen-processing machinery
CGA: Cancer-germline antigens
CTL: Cytotoxic T lymphocyte
DGE: Differential gene expression
EM: Effector memory
FC: Fold change
FFPE: Formalin-fixed paraffin-embedded
gMFI: Geometric mean fluorescence intensity
GSEA: Gene set enrichment analysis
HDAC: Histone deacetylase
HDACi: HDAC inhibitor
i.p.: Intraperitoneally
MDSC: Myeloid-derived suppressor cell
MHC-I / MHC-II: Major histocompatibility class I / class II
ns: Not significant
p.o.: per os, orally
PBMC: Peripheral blood mononuclear cells
PD-1: Programmed cell death protein-1
PD-L1: Programmed cell death ligand-1
RE: Response
RT: Room temperature
s.c.: Subcutaneously
SD: Standard deviation
TIME: Tumor immune microenvironment
TMB: Tumor mutational burden
TPM: Transcripts per million
Treg: Regulatory T cell
