# Supplementary material for: Domatinostat favors the immunotherapy response by modulating the tumor immune microenvironment (TIME)
Source: J Immunother Cancer. 2019 Nov 8;7:294. doi: 10.1186/s40425-019-0745-3 (PMC6839078; doi:10.1186/s40425-019-0745-3)
Supplement: Supplementary file 1 — Additional file 1: Supplementary methods. (DOCX 56 kb) [file 40425_2019_745_MOESM1_ESM.docx]

Additional file 1: Supplementary Methods

## HDAC profiling

Functional activities of HDACs were assessed by using acetylated 7-amino-4-methylcoumarin (AMC)-labeled fluorogenic substrates: for HDAC1, -2, -3, -6, -10, and -11: a peptide from p53 residues 379-382 (RHKK(Ac)-AMC); for HDAC4, -5, -7, and -9: an HDAC class IIa substrate (trifluoroacetyl-lysine-AMC) and for HDAC8 a peptide from p53 residues 379-382 (RHK(Ac)K(ac)AMC). Deacetylated products were fluorescently measured (excitation/emission, 360/460 nm). K_i_ values, which are independent of experimental conditions, were calculated by using the Cheng-Prusoff equation: K_i_ = IC_50_ / ((1 + ([S] / K_m_)) [1], where IC50 is the inhibitor concentration needed to reduce the activity of an enzyme by half, [S] is the fixed substrate concentration and K_m_ is the substrate concentration at which the enzyme activity is half-maximal.

## Cell lines and culture

Human malignant melanoma cell lines A375, HMCB and MEWO were obtained from ATCC (CRL-1619, CRL-9607) and CLS (300285). The murine colon carcinoma cell line CT26.WT was from ATCC (CRL-2638). A375 cells were cultured in DMEM with high glucose and L-glutamine (PAN). HMCB and MEWO were cultured in Eagle’s MEM (PAN). CT26 cells were cultured in RPMI-1640 (PAN) supplemented with 10% fetal bovine serum (FBS, Gibco). Cell lines were regularly tested for the absence of mycoplasma and authenticated by STR profiling. Peripheral blood mononuclear cells (PBMCs) from healthy human donors were purified by centrifugation using Leucosep tubes (Greiner) according to the manufacturer’s instructions and washed two times in phosphate-buffered saline (PBS) supplemented with 1% FBS. Residual erythrocytes were lysed (155 mM NH_4_Cl, 10 mM KHCO_3_, 0.1 mM EDTA) and cells seeded in RPMI-1640 GlutaMAX supplemented with 10% FBS (both Gibco), 100 U/mL penicillin, 100 mg/mL streptomycin (PAA) and 2 mM L-glutamine (PAA).

For compound treatment, cells were seeded 1 d before. Domatinostat was dissolved in 100% DMSO, diluted and applied at the indicated concentrations with a final solvent concentration of 0.1% DMSO. Recombinant IFN-γ (Peprotech) was used at 10 ng/mL. The TLR agonist R848 (Sigma-Aldrich) was used at 3 µM.

## Flow cytometry analysis of tumor cell lines

Adherent cells were detached with Detachin reagent (BioCat, T100106) and resuspended in MACS buffer (PBS EDTA pH 7.5, Lonza) supplemented with 3% FBS. Cells were incubated with fluorophore-coupled antibodies in MACS buffer for 30 min on ice. Cells were washed once in MACS buffer and analyzed on a MACSQuant Analyzer 10 (Miltenyi Biotec). Before measurement, propidium iodide (Miltenyi Biotec, 1.5 µg/mL) or DAPI (Miltenyi Biotec, 0.1 µg/mL) was added to each sample for live-dead discrimination. Data were evaluated with FlowLogic (Inivai). Quantitative expression of selected markers is presented as median fluorescence intensity (FI) of viable singlet cells.

## Antibodies

Antibodies for flow cytometry analyses of tumor tissue

| **Antibody** | **Vendor** | **Catalogue number** |
| --- | --- | --- |
| CD3 | BD Biosciences | 561388 |
| CD4 | Miltenyi Biotec | 130-102-444 |
| CD8a | Miltenyi Biotec | 130-109-251 |
| CD11b | Miltenyi Biotec | 130-091-241 |
| CD44 | Miltenyi Biotec | 130-110-085 |
| CD45 | Miltenyi Biotec | 130-110-665 |
| CD62L | BD Biosciences | 564109 |
| CD69 | Miltenyi Biotec | 130-115-461 |
| CD103 | Miltenyi Biotec | 130-108-184 |
| CD223 (=LAG3) | Miltenyi Biotec | 130-111-329 |
| CD279 (=PD-1) | BD Biosciences | 744546 |
| FOXP3 | Miltenyi Biotec | 130-093-014 |
| GITR | Miltenyi Biotec | 130-116-428 |
| GR1 | Miltenyi Biotec | 130-102-141 |
| IgG1 | Miltenyi Biotec | 130-92-212 |
| IgG2 | BD Biosciences | 560457 |
| IgG2a | Miltenyi Biotec | 130-102-650 |
| IgG2b | Miltenyi Biotec | 130-102-656 |
| Ki-67 | Miltenyi Biotec | 130-100-330 |
| LY-6C | Miltenyi Biotec | 130-111-784 |
| LY-6G | Miltenyi Biotec | 130-102-296 |
| MHC class I | eBioscience | 11-5999-82 |
| MHC class II | Miltenyi Biotec | 130-112-233 |
| REA control | Miltenyi Biotec | 130-104-630 |
| TIGIT | BD Biosciences | 565168 |
| Viability 405/452 | Miltenyi Biotec | 130-110-205 |

Antibodies for flow cytometry analyses of tumor cell lines

| **Antibody** | **Vendor** | **Catalogue number** |
| --- | --- | --- |
| HLA-ABC-APC (human) | Miltenyi Biotec | 130-101-467 |
| H-2Kd/H-2Dd-APC (mouse) | Miltenyi Biotec | 130-107-895 |
| REA control-APC (isotype control) | Miltenyi Biotec | 130-104-615 |

Antibodies for IHC analyses

| **Antibody** | **Vendor** | **Catalogue number** |
| --- | --- | --- |
| CD3 (rabbit) | Abcam | ab5690 |
| CD8a (rat) | Affymetrix | 14-0808 |
| Goat anti-rabbit IgG-HRP | Leica | DS9800 |
| Goat anti-rat IgG-HRP | Vector | MP7444-15 |
| Rabbit IgG (isotype control) | Vector | I-1000 |
| Rat IgG (isotype control) | Vector | I-4000 |

## IFN-γ detection by enzyme-linked immunosorbent assay (ELISA)

IFN-γ secretion by human PBMCs was analyzed using a colorimetric ELISA (Human IFN-γ ELISA Set, BD) according to the manufacturer’s instructions, and optical density (OD) was measured with an absorbance plate reader (Tecan Sunrise) at 450 nm. OD values were blank-corrected. Comparable cell numbers between wells were verified using the ViaLight Plus Cell Proliferation and Cytotoxicity BioAssay Kit (Lonza) according to the manufacturer’s instructions.

## Bioinformatic analyses

For pathway analysis, differentially expressed genes (DEGs) were first filtered for significance (adjusted *P*-value < 0.05) and log2(fold change) (FC) threshold >|1|. Filtered DEGs were then analyzed for enriched pathways with Enrichr (<http://amp.pharm.mssm.edu/Enrichr/>) [2].

The type and density of tumor-infiltrating immune cells in human tumor samples were estimated by deconvolution of RNA-seq data using quanTIseq with default parameters (<https://icbi.i-med.ac.at/software/quantiseq/doc/index.html>) [3].

## Gene expression analysis of CT26 tumors and cell lines by qPCR

Equal amounts of RNA were reverse transcribed into cDNA using the GoScript Reverse Transcriptase with random primers (Promega). Gene expression was measured in real time in the LightCycler LC96 (Roche) by using SYBR Green, GoTaq qPCR Master Mix (Promega), and specific primers as listed in the table below. Relative gene expression was calculated with the ΔΔCq method, and *Actb* was used as a reference gene.

qPCR primer sequences

| **Gene** | **Forward** | **Reverse** |
| --- | --- | --- |
| *Actb* | AGATCAAGATCATTGCTCCTCCT | ACGCAGCTCAGTAACAGTCC |
| *Ccl5* | CTCACCATATGGCTCGGACACC | GCGGTTCCTTCGAGTGACAA |
| *Ccl8* | AAGCTGAAGATCCCCCTTCG | TGCTTGGTCTGGAAAACCACA |
| *H2-Aa* | AGCTTCTTCGTCAACCGTGA | TCTCAGGTTCCCAGTGTTTCAG |
| *H2-Eb1* | CACGGTCGAGTGGAAAGCA | GTAGATGAACAGCCCCGCTC |
| *H2-M2* | TTTAAAGGATCCCACTCCTTGCGG | TCTGTTCCATCCAAGGCACTC |
| *H2-M11* | TGTTACCGAGGCTCTCTTCCT | GTGGGTACCATCTGGATGACTG |
| *Ido1* | AGGATGCGTGACTTTGTGGA | TCCCAGACCCCCTCATACAG |
| *Ifng* | CGGCACAGTCATTGAAAGCC | TGTCACCATCCTTTTGCCAGT |

# References:

1. Cheng Y, Prusoff WH. Relationship between the inhibition constant (K1) and the concentration of inhibitor which causes 50 per cent inhibition (I50) of an enzymatic reaction. Biochem Pharmacol. 1973;22:3099–108.

2. Kuleshov M V., Jones MR, Rouillard AD, Fernandez NF, Duan Q, Wang Z, et al. Enrichr: a comprehensive gene set enrichment analysis web server 2016 update. Nucleic Acids Res. 2016;44:W90–7.

3. Finotello F, Mayer C, Plattner C, Laschober G, Rieder D, Hackl H, et al. Molecular and pharmacological modulators of the tumor immune contexture revealed by deconvolution of RNA-seq data. Genome Med. Genome Medicine; 2019;11:34.
