# Supplementary material for: Domatinostat favors the immunotherapy response by modulating the tumor immune microenvironment (TIME)
Source: J Immunother Cancer. 2019 Nov 8;7:294. doi: 10.1186/s40425-019-0745-3 (PMC6839078; doi:10.1186/s40425-019-0745-3)
Supplement: Supplementary file 2 — Additional file 2: Figure S1. In vitro effects of domatinostat on antigen presentation by human melanoma and murine CT26 cells. Figure S2. Immune cell profiling of mouse syngeneic CT26 tumors. Figure S3. In vivo effects of domatinostat on gene expression in murine syngeneic CT26 tumors. Figure S4. Domatinostat increases MHC class II expression in MDSCs in the syngeneic C38 tumor model. Figure S5. Phenotype of immune checkpoint-positive cells after domatinostat or anti-PD-1 treatment in the syngeneic C38 tumor model. Figure S6. Gene expression analysis of patient-derived domatinostat-treated melanoma biopsies. Figure S7. Domatinostat has no direct effect on IFN-γ expression. Table S1. Patient characteristics (SENSITIZE, cohort 1). (DOCX 2645 kb) [file 40425_2019_745_MOESM2_ESM.docx]

Additional file 2: Supplementary figures and tables

[**Supplementary figure S1:** In vitro effects of domatinostat on antigen presentation by human melanoma and murine CT26 cells. 2](#_Toc17102543)

[**Supplementary figure S2:** Immune cell profiling of mouse syngeneic CT26 tumors. 4](#_Toc17102544)

[**Supplementary figure S3:** In vivo effects of domatinostat on gene expression in murine syngeneic CT26 tumors 5](#_Toc17102545)

[**Supplementary figure S4:** Domatinostat increases MHC class II expression in MDSCs in the syngeneic C38 tumor model. 7](#_Toc17102546)

[**Supplementary figure S5:** Phenotype of immune checkpoint-positive cells after domatinostat or anti-PD-1 treatment in the syngeneic C38 tumor model. 8](#_Toc17102547)

[**Supplementary figure S6:** Gene expression analysis of patient-derived domatinostat-treated melanoma biopsies. 9](#_Toc17102548)

[**Supplementary figure S7:** Domatinostat has no direct effect on IFN-γ expression. 10](#_Toc17102549)

[**Supplementary table S1:** Patient characteristics (SENSITIZE, cohort 1) 11](#_Toc17102562)

[References 12](#_Toc17102571)


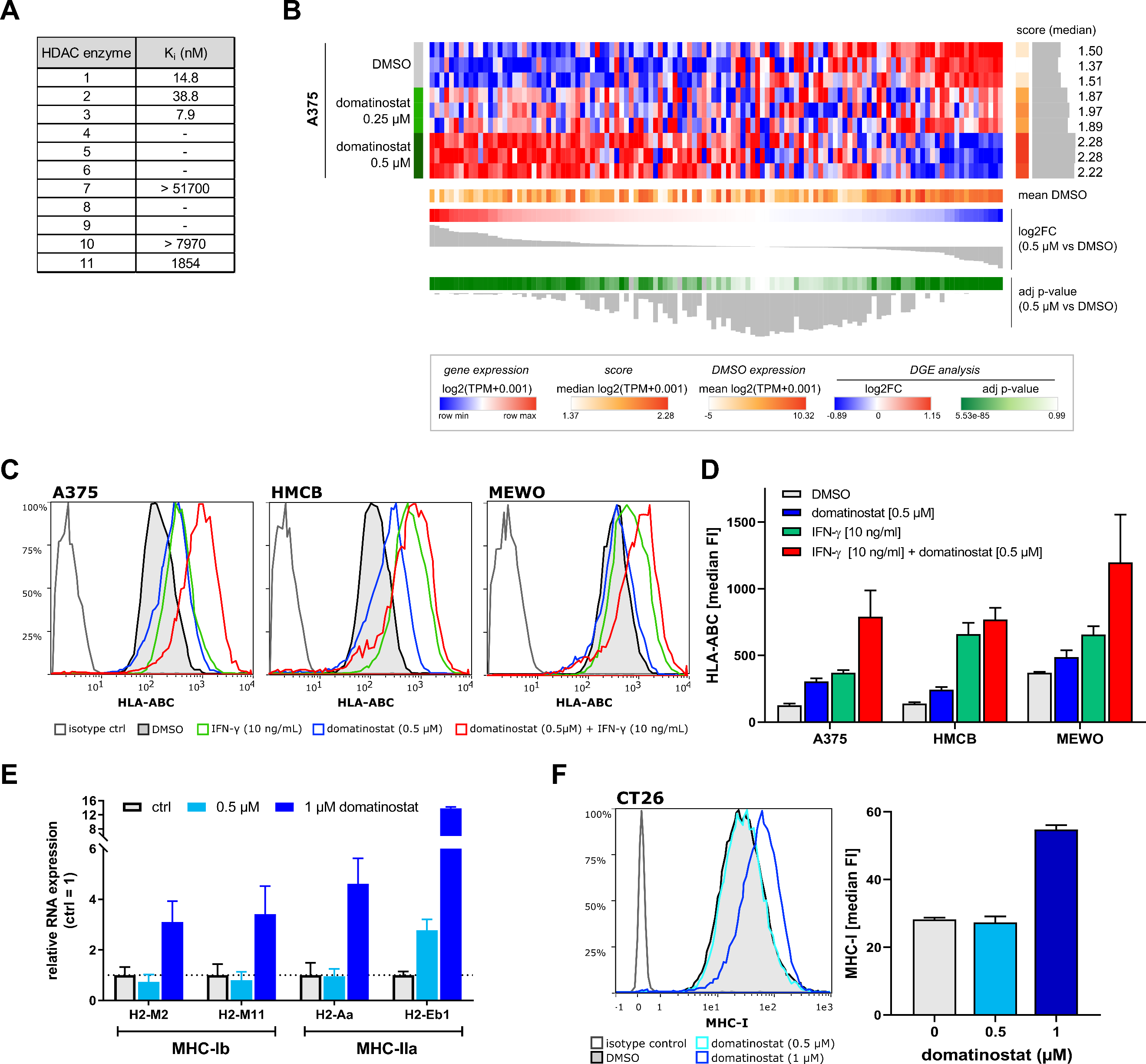


Supplementary figure S1: In vitro effects of domatinostat on antigen presentation by human melanoma and murine CT26 cells.

**a,** Inhibitory constant (Ki) values of domatinostat on human recombinant HDAC enzymes reflecting class I selectivity. **b,** Heatmap of CGA genes (CTdatabase <http://www.cta.lncc.br/>) [1] expressed in A375 melanoma cells after incubation with domatinostat (0.25 µM, 0.5 µM) or DMSO (control) for 24 h; low-expressed genes (mean log2(TPM+0.001) < -5) were filtered out; expression scores per sample were calculated by median log2(TPM+0.001); genes were sorted by their log2-fold change (FC) (0.5 µM versus DMSO) from DGE analysis, and log2FC is illustrated with corresponding adjusted p-values below the heatmap. **c, d,** Flow cytometric analysis of MHC class I (HLA-ABC) cell surface expression in melanoma cell lines A375, HMCB and MEWO following incubation with domatinostat (0.5 µM), IFN-γ (10 ng/mL), their combination or DMSO (control) for 72 h; **c,** representative histograms; **d,** quantification of expression by median fluorescence intensity, (FI, mean + SD, n=3). **e,** Gene expression analysis of MHC class I and II gene expression by qPCR in murine CT26 cells after incubation with domatinostat (0.5 µM, 1 µM) or DMSO (ctrl, control) for 48 h (mean + SD, n=3). **f,** Flow cytometric analysis of MHC class I expression in murine CT26 cells after incubation with domatinostat (0.5 µM, 1 µM) or DMSO for 72 h; left: representative histogram; right: quantification by median fluorescence intensity (FI, mean + SD, n=3).


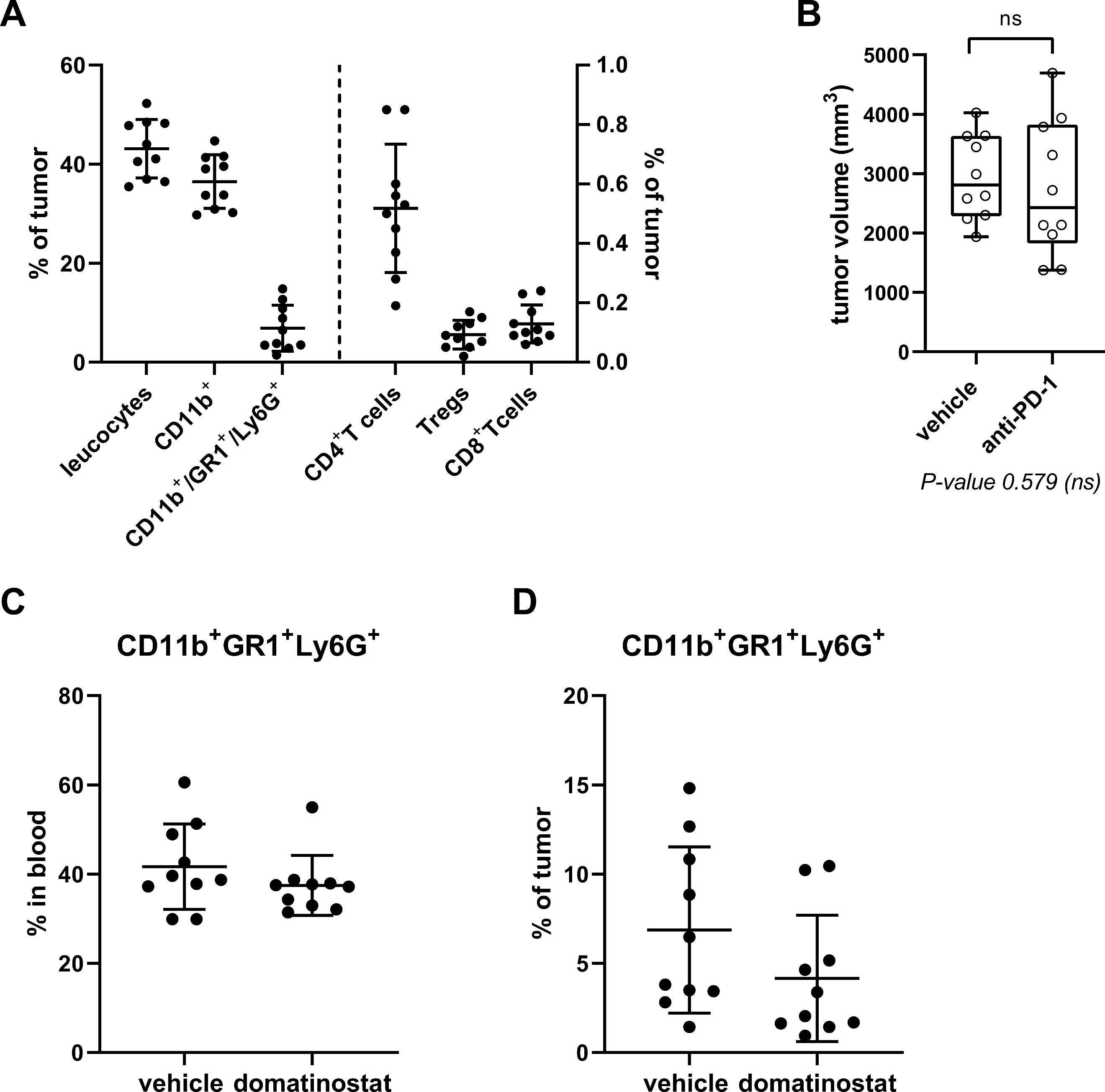


Supplementary figure S2: Immune cell profiling of mouse syngeneic CT26 tumors.

**a,** Composition of immune cell subpopulations in CT26 tumors derived from vehicle-treated animals (n=10). **b,** Tumor volumes of CT26 animals (n=10 per group) treated with anti-PD-1 or vehicle. **c, d,** Proportion of MDSCs (CD11b^+^GR1^+^Ly6G^+^) in blood (**c**) and tumors (**d**) of the CT26 syngeneic tumor model after treatment with domatinostat or vehicle as in Fig. 1.

**a, c, d,** Mean ± SD showing all data points. **b,** Box plots showing all data points (whiskers min to max). P-values: Mann-Whitney test, two-tailed; ns, not significant.


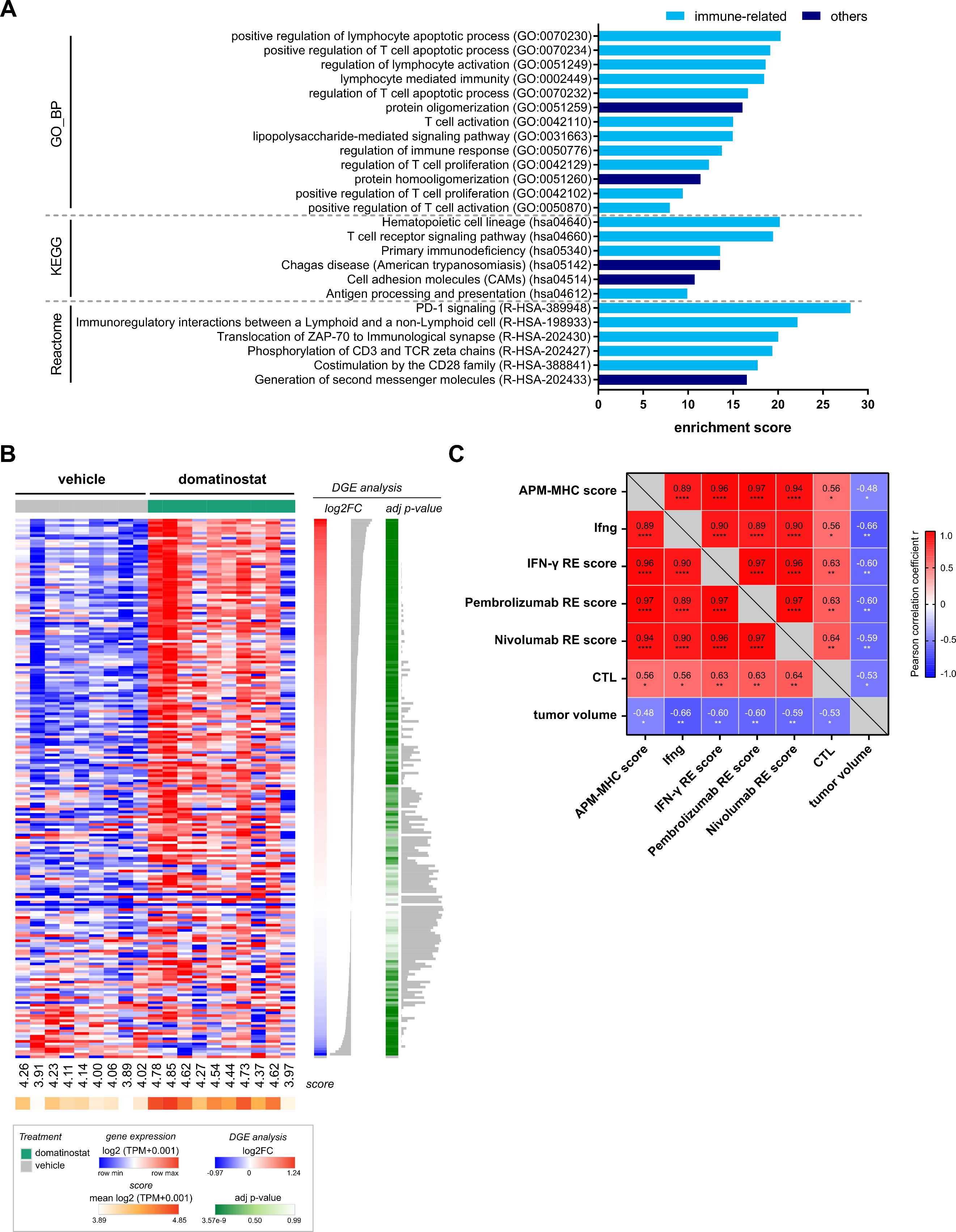


Supplementary figure S3: In vivo effects of domatinostat on gene expression in murine syngeneic CT26 tumors

A total of 1x10^6^ CT26 cells were inoculated s.c. into BALB/c mice (n=10 per group); when tumor volumes reached 150 mm^3^, animals were treated with domatinostat (20 mg/kg twice daily) or vehicle; after the end of treatment, tumors were harvested, and gene expression was analyzed by RNA-seq (see also Fig. 2). **a,** Pathway analysis of domatinostat-induced genes (log2-fold change (FC) >1, adjusted *P*-value <0.05) by Enrichr [2]. Significant results for gene ontology biology processes (GO-BP), Reactome, and KEGG pathways are shown. Domatinostat led to enrichment of immune-related gene sets (20/25). **b,** Heatmap of IFN-γ response signature gene expression (MSigDB hallmark gene set) in domatinostat- versus vehicle-treated animals (genes sorted by log2(fold change) (FC) of the DGE analysis; adjusted *P*-values listed for each gene). Expression scores for each sample were calculated by mean log2(TPM+0.001) (see Fig. 2d, e). **c,** Heatmap of Pearson correlation coefficients of gene expression scores for the APM/MHC signature, the *Ifng* gene, and the IFN-γ, pembrolizumab and nivolumab response (RE) signatures (see Fig. 2), as well as overall CTL numbers (% CTL of tumor) and tumor volumes (see Fig. 1b,d). Pearson correlation coefficient r and significance of corresponding *P*-values (two-tailed) are listed. *, *P* <0.05; **, *P* <0.01; ***, *P* <0.001; ****, *P* <0.0001.


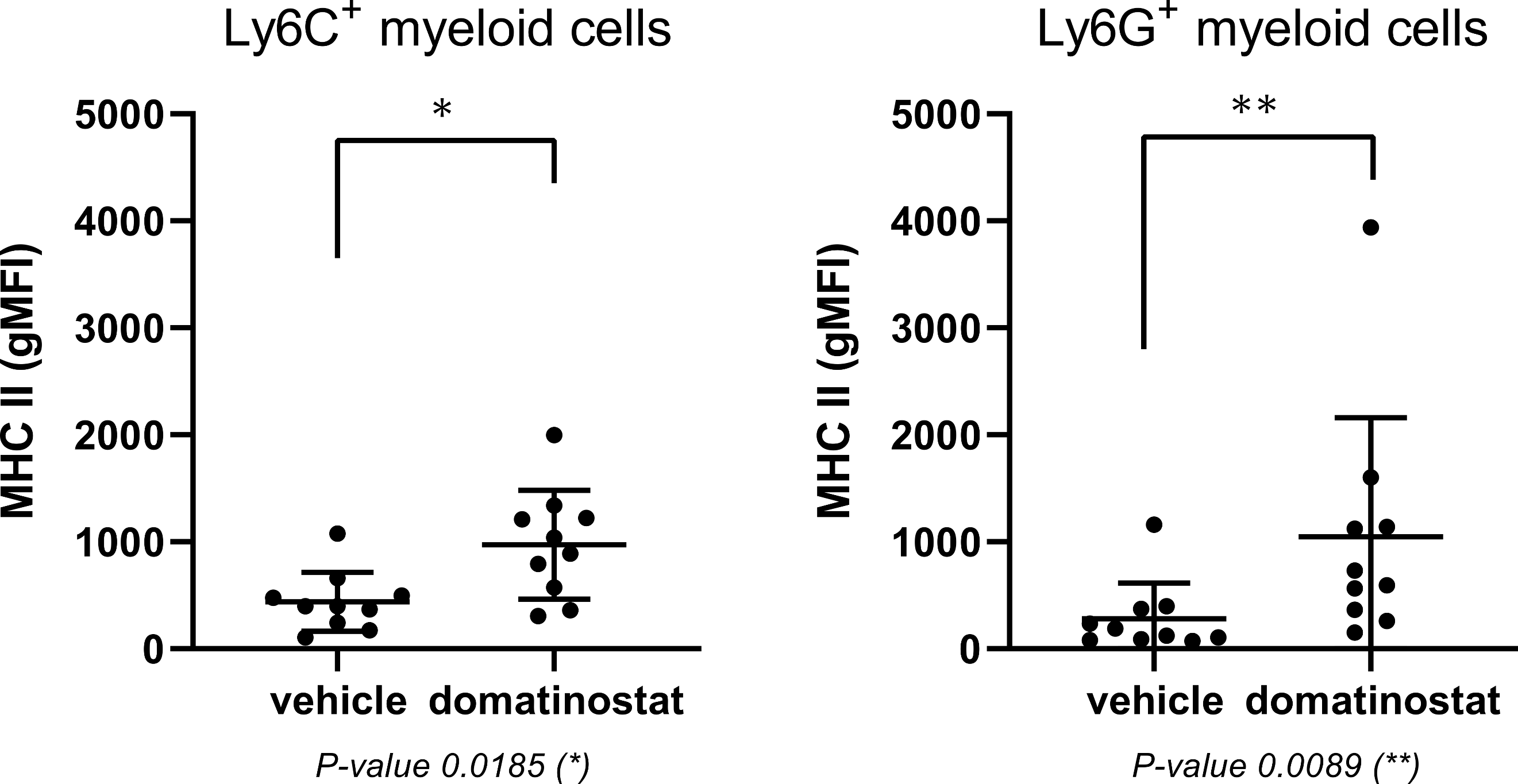


Supplementary figure S4: Domatinostat increases MHC class II expression in MDSCs in the syngeneic C38 tumor model.

C38 tumor fragments were inoculated s.c. into C57BL/6J mice; when tumor volumes reached 150 mm^3^, animals (n=10 per group) were treated with 20 mg/kg domatinostat or vehicle twice daily; tumors were harvested for analysis of cell populations by flow cytometry after 18 treatment days (see also Fig. 4). Mean MHC class II expression on CD45^+^CD11b^+^ cells (MDSCs) expressing Ly6C (left) or Ly6G (right). Note that in the vehicle group, MHC class II expression was lower on MDSCs than on M1 macrophages (Fig. 4b), indicating the immature phenotype of MDSCs.

Mean ± SD showing all data points. gMFI, geometric mean fluorescence intensity. P-value: Mann-Whitney test, two-tailed. *, P <0.05; **, P <0.01.


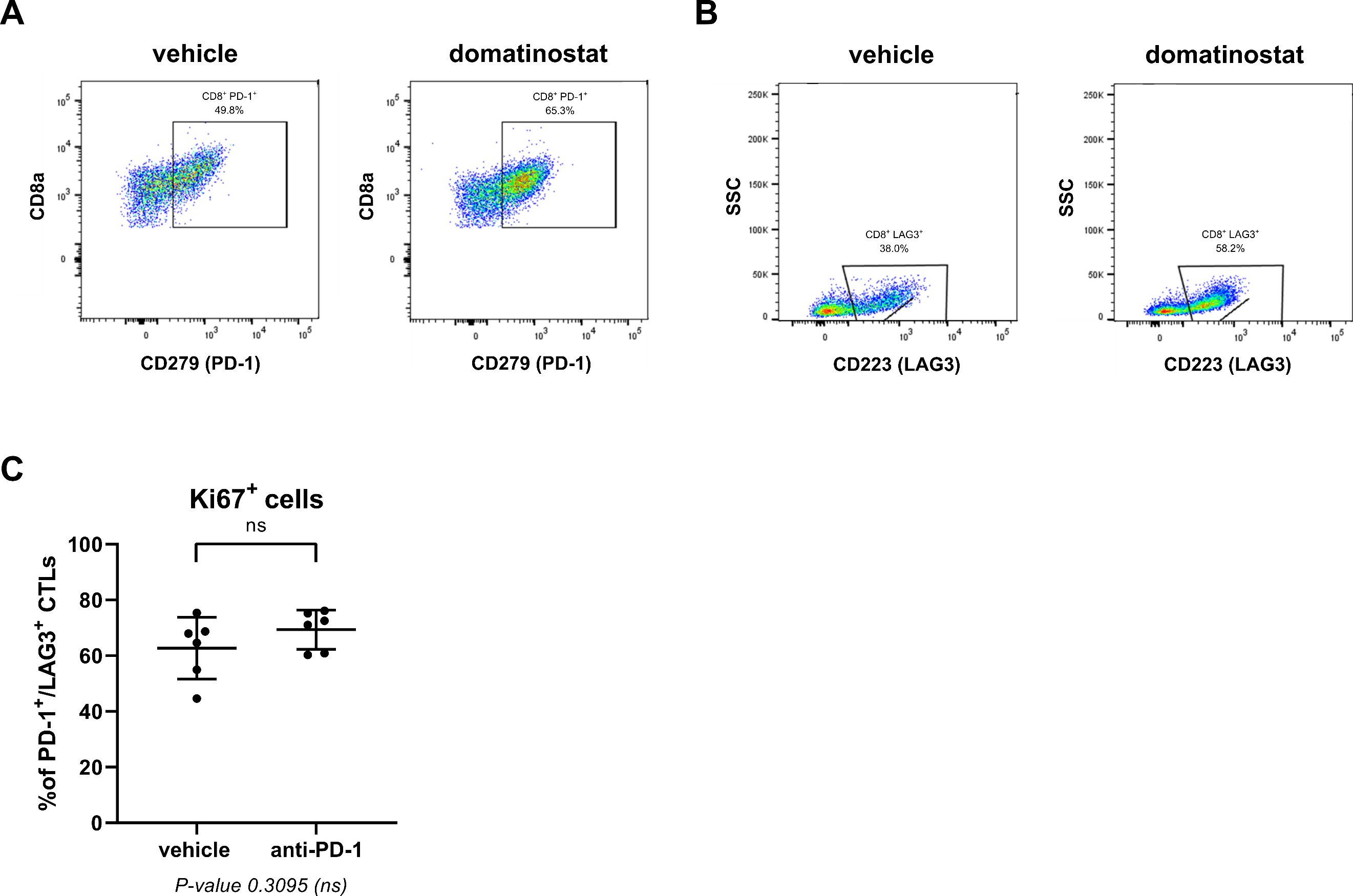


Supplementary figure S5: Phenotype of immune checkpoint-positive cells after domatinostat or anti-PD-1 treatment in the syngeneic C38 tumor model.

C38 tumor fragments were inoculated s.c. into C57BL/6J mice; when tumor volumes reached 150 mm^3^, animals (n=10 per group) were treated with 20 mg/kg domatinostat twice daily (**a, b**), 10 mg/kg anti-PD-1 twice a week (**c**) or vehicle; tumors were harvested for analysis of cell populations by flow cytometry after 9 (**a, b**; n=6) or 18 treatment days (**c,** n=10). **a, b,** Representative dot plots illustrating expression levels of PD-1 (**a**) and LAG3 (**b**) on intratumoral CD8^+^ CTLs of vehicle- and domatinostat-treated animals. Of note, the mean intensity of the PD-1^+^/LAG3^+^ subpopulation remained unchanged while its percentage increased. **c,** Proportion of Ki67^+^ (proliferation marker) cells in the PD-1^+^/LAG3^+^ subpopulation of CTLs upon anti-PD-1 treatment.

**c,** Mean ± SD showing all data points. P-value: Mann-Whitney test, two-tailed; ns, not significant.


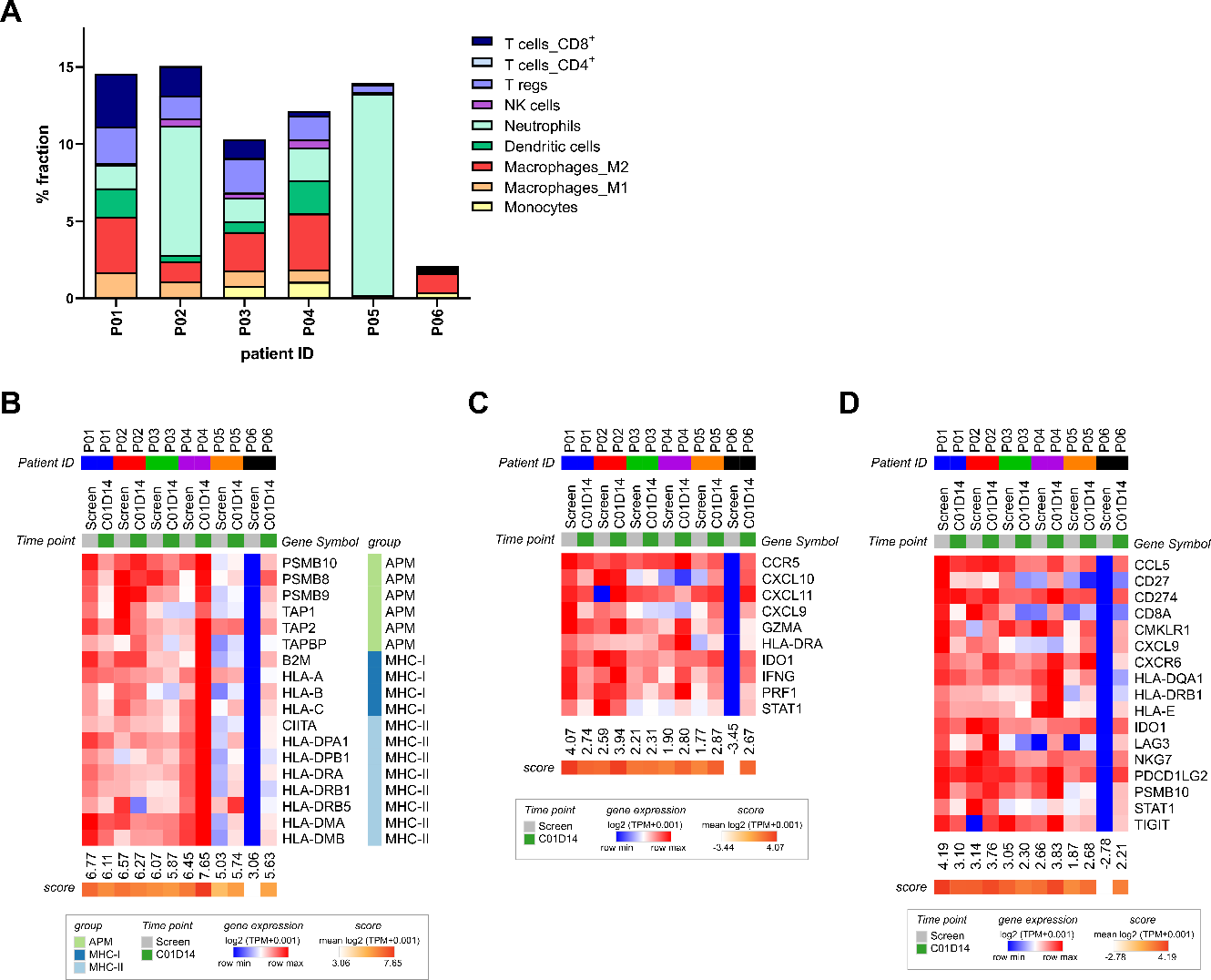


Supplementary figure S6: Gene expression analysis of patient-derived domatinostat-treated melanoma biopsies.

Patients (n=6; P01-P06) with advanced cutaneous melanoma treated with 100 mg domatinostat once daily in a phase I/II clinical trial (SENSITIZE trial: NCT03278665) were subjected to biopsy of tumor lesions before (screen, baseline) and after 14 days of treatment (C01D14) for analysis of gene expression (RNA-seq). **a,** Composition of different immune cells within baseline tumor samples (pretreatment) in individual patients estimated by quanTIseq [3]. Patients were sorted according to the fraction of CD8^+^ T cells. **b-d,** Heatmaps of gene expression data of the APM/MHC (**b**), 10-gene IFN-γ-related (**c**) [4] and pembrolizumab response signatures (**d**) [4]; comparison of scores is provided in Fig. 7.


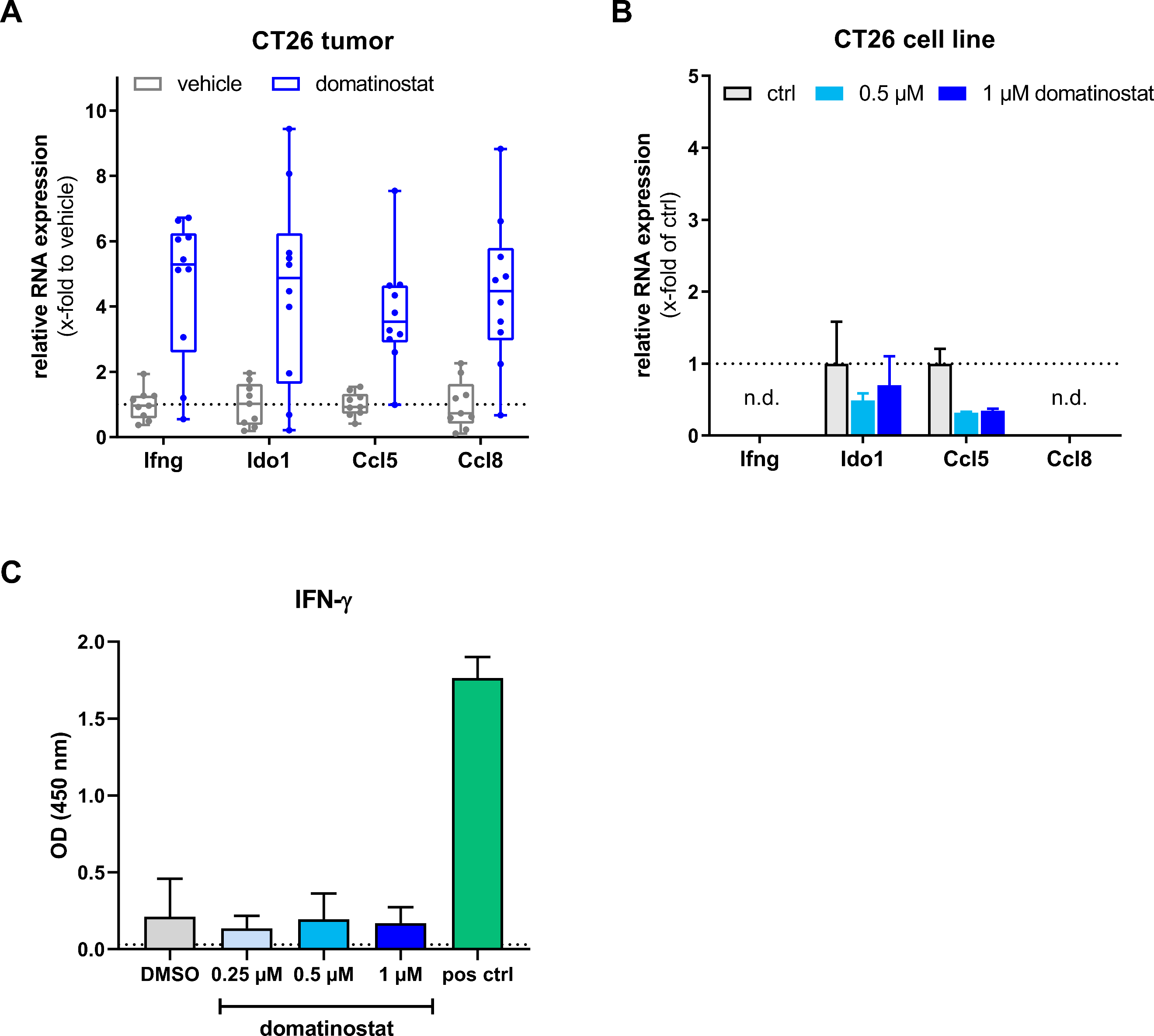


Supplementary figure S7: Domatinostat has no direct effect on IFN-γ expression.

**a, b,** Gene expression analysis of *Ifng* and IFN-γ target genes (*Ido1*, *Ccl5*, *Ccl8*) by qPCR. **a,** End-of-treatment tumors from the syngeneic CT26 tumor model with and without domatinostat treatment (n=10 per group) as in Figs. 1 and 2*.* **b,** CT26 tumor cells after *in vitro* incubation with domatinostat (0.5 µM, 1 µM) or DMSO (ctrl, control) for 48 h. **c,** IFN-γ secretion of peripheral blood mononuclear cells (PBMCs) with and without domatinostat treatment at the indicated concentrations for 48 h. TLR agonist R848 was used as a positive control.

**a,** Box plots showing all data points (whiskers: min to max). **b,** Mean + SD, n=3; n.d. = not detectable. **c,** Bar charts showing mean + SD, n=6.

Supplementary table S1: Baseline characteristics of patients treated in the first-dose cohort of the SENSITIZE clinical trial (NCT03278665)

The following table summarizes the baseline characteristics of patients (n=6, P01-P06) whose tissue samples were analyzed by RNA-seq before and after 14 days of treatment with 100 mg domatinostat monotherapy once daily during the SENSITIZE clinical trial (NCT03278665), cohort 1. No significant differences in pharmacokinetics were observed (data not shown).

Supplementary table S1: Patient characteristics (SENSITIZE, cohort 1)^1^

| **Characteristics** | **n** |
| --- | --- |
| Number of patients | 6 |
| AJCC stage IV, M1a / M1b / M1c | 0 / 1 / 5 |
| Prior immune checkpoint inhibitor  Anti-CTLA4 + anti-PD-1 / anti-PD-1 / both^2^ | 2 / 1 / 3 |
| Primary refractory / nonresponding^3^ | 3 / 3 |

*^1^ preliminary, unconfirmed data (trial ongoing)*

*^2^ both: consecutive treatment with anti-CTLA-4 or combined anti-CTL-4+anti-PD-1 and anti-PD‑1 antibodies*

*^3^ nonresponding: stable disease or progressive disease as the best response to prior anti-PD-1 treatment*

# References

1. Almeida LG, Sakabe NJ, de Oliveira AR, Silva MCC, Mundstein AS, Cohen T, et al. CTdatabase: A knowledge-base of high-throughput and curated data on cancer-testis antigens. Nucleic Acids Res. 2009;37:2007–10.

2. Kuleshov M V., Jones MR, Rouillard AD, Fernandez NF, Duan Q, Wang Z, et al. Enrichr: a comprehensive gene set enrichment analysis web server 2016 update. Nucleic Acids Res. 2016;44:W90–7.

3. Finotello F, Mayer C, Plattner C, Laschober G, Rieder D, Hackl H, et al. Molecular and pharmacological modulators of the tumor immune contexture revealed by deconvolution of RNA-seq data. Genome Med. Genome Medicine; 2019;11:34.

4. Ayers M, Lunceford J, Nebozhyn M, Murphy E, Loboda A, Kaufman DR, et al. IFN- γ – related mRNA profile predicts clinical response to PD-1 blockade. J Clin Invest. 2017;127:2930–40.
